# Outcomes of acute exacerbations in COPD in relation to pre-hospital oxygen therapy

**DOI:** 10.3402/ecrj.v2.27283

**Published:** 2015-05-11

**Authors:** Thomas J. Ringbaek, Jakob Terkelsen, Peter Lange

**Affiliations:** 1 Department of Respiratory Medicine, University Hospital of Copenhagen, Hvidovre, Denmark; 2 Section of Social Medicine, Department of Public Health, University of Copenhagen, Denmark

**Keywords:** COPD, respiratory acidosis, hypercapnic respiratory failure, oxygen therapy, ambulance, pre-hospital care, arterial blood gases, survival, exacerbation

## Abstract

**Background:**

Pre-hospital, high-concentration oxygen therapy during acute exacerbation of chronic obstructive pulmonary disease (AECOPD) has been associated with increased mortality. Recent COPD guidelines have encouraged titrated oxygen therapy with a target saturation range of 88–92%. Oxygen therapy leading to saturation above 92% is defined as ‘inappropriate oxygen therapy’.

**Objectives:**

To examine the frequency of inappropriate oxygen therapy and whether inappropriate oxygen therapy in the ambulance in an urban area with short transit time to hospital was associated with poor outcome.

**Methods:**

In an audit of 405 consecutive patients with AECOPD arriving by ambulance to Hvidovre Hospital, details of transit time, oxygen administration, saturation, and arterial blood gases were registered. Outcomes were respiratory acidosis, need of supported ventilation, length of hospitalisation, and in-hospital mortality.

**Results:**

Only 15 patients were not treated with oxygen and information on oxygen flow was missing in seven patients and on saturation on one patient. Altogether, 352 (88.7%) of 397 patients received inappropriate oxygen therapy. Patients on ‘inappropriate oxygen therapy’ (saturation ≥92%) had a high frequency of respiratory acidosis at hospital admission, 108 (33.5%) of 324 patients, length of stay was on average 5.1 days, 12.5% of the patients needed ventilatory support, and in-hospital mortality was 3.4%.

**Conclusion:**

The majority of patients with AECOPD received inappropriate oxygen therapy in the ambulance, but their need of ventilatory support, length of stay, and mortality were low. Randomised studies are needed to clarify the optimal pre-hospital oxygen therapy.

Acute exacerbation in COPD (AECOPD) is a major cause of acute hospitalisation (1). COPD exacerbations are often accompanied by respiratory failure with high in-hospital mortality (2).

Acute hypoxaemia is immediately life threatening, but over-oxygenation in patients at risk of type 2 respiratory failure (hypercapnia) is also dangerous because it can lead to respiratory acidosis, organ dysfunction, coma, and death (3).

Although the risk of high-concentration oxygen (HCO) therapy has been known for more than 50 years (4), HCO therapy in patients with AECOPD arriving by ambulance to emergency department (ED) is still common (5, 6). This problem was recently demonstrated in a randomised study, showing that COPD patients brought by ambulance with HCO therapy had significantly higher mortality compared with patients treated with titrated oxygen (7). Subsequently, COPD guidelines have encouraged titrated oxygen therapy with a targeted saturation range of 88–92% in those at risk of hypercapnic respiratory failure including COPD patients (8–10). Oxygen therapy leading to saturation above 92% in COPD patients has been defined as ‘inappropriate oxygen therapy’ (11). Only few studies have evaluated pre-hospital oxygen therapy and consequences for arterial blood gas tensions and requirement of assisted ventilation (6, 7, 11), and to our knowledge, the frequency of inappropriate oxygen therapy has not been reported previously.

The aims of our study were to determine whether the use of pre-hospital oxygen in AECOPD was in accordance with the international guidelines (appropriate oxygen therapy), and whether ‘inappropriate oxygen therapy’ administrated by the ambulance crew in an urban area with expected short transit time was associated with poor outcome.

## Methods

We undertook a retrospective audit of all patients transported by ambulance and admitted to Hvidovre Hospital with an exacerbation in COPD between 1 January 2012 and 30 August 2012. Patients were identified by a primary diagnosis of COPD exacerbation (ICD code J440 or J441) at discharge (including those who died at hospital).

It has been suggested that frequency of inappropriate oxygen therapy could be reduced, if COPD patients sensitive to oxygen therapy are equipped with alert cards, which they could show to the ambulance and ED staff (10, 12). Oxygen alert cards were not used in our study.

The ambulance case records provided information on transit time (time from arrival at patient's home to arrival at hospital), pre-hospital oxygen therapy, and pulse oximetry. From the patients’ hospital file, we achieved information on blood gases and pH from the first arterial puncture on arrival in the ED, non-invasive ventilation (NIV), invasive ventilation, and death. We registered assisted ventilation within the first 24 h of the admission and death within the first 5 days of hospital admission and during hospital admission.

Hvidovre Hospital is a large university hospital, situated in the south part of Copenhagen, serving approx. 250,000 inhabitants.

Danish guidelines recommend an initial oxygen flow of 3–5 L/min (equivalent to 40%) via nasal cannula or 7–10 L/min through a mask and then adjusted with a targeted saturation range of 90–93% (87–90% if the patient becomes drowsy). There should be artery puncture within half an hour after starting oxygen therapy to ensure adequate PaO_2_ without CO_2_ retention and acidosis. NIV and invasive ventilation should be considered in patients with respiratory acidosis (P_a_CO_2_>6.0 kPa and pH<7.35) who did not normalise their ABG after the initial treatment (13).

## Statistics

Analyses were performed with the statistical package for the social sciences (SPSS) ver. 13.0 (SPSS Inc., Chicago, USA). The chi-squared, two sample *t*-tests and Mann–Whitney U tests were used as appropriate to compare differences between groups. To evaluate the association between transit time and outcomes (respiratory acidosis, assisted ventilation, length of stay, and in-hospital mortality), transit time was divided into tertiles. A two-sided *p* value of <0.05 was considered significant.

## Results

In total, 700 patients were admitted to hospital with an exacerbation in COPD in the observation period. One hundred and twenty nine patients were referred from other hospitals, other departments, or from an outpatient clinic, and 153 were not brought by ambulance. Four hundred and eighteen patients were transported by ambulance. In 12 cases, the medical records were missing, and one patient who was intubated at home before transportation to the hospital was excluded from the study. Patients’ characteristics and data from the ambulance records in 405 cases are shown in Table 1.

**Table 1:** Patient characteristics and pre-hospital transit time, administration of oxygen, and oxygen saturation measured in the ambulance immediately before arrival to the emergency department (ED) in patients who were hospitalised with an exacerbation in COPD

			Arterial blood gases in the ED		
				
		All, *N*=405	Yes, *N*=368	No, *N*=37	*p*
Age, years (SD)		71.6 (11.3)	71.6 (10.9)	71.5 (15.0)	0.96
Home oxygen therapy		43 (10.6%)	40 (10.9%)	3 (8.1%)	0.78
Males		181 (44.7%)	163 (44.3%)	18 (48.6%)	0.62
Pre-hospital	Transit time (min)	30.5 (9–62)	30.7 (9–62)	27.9 (13–60)	0.11
	Oxygen flow on mask (L/min); *n*=356	8.3 (0–15)	8.3 (0–15)	8.0 (0–15)	0.88
	Oxygen flow nasal (L/min); *n*=33	3.3 (1–6)	3.1 (1–5)	3.8 (2–6)	0.30
	Oxygen saturation at arrival (%)	96.8 (61–100)	96.8 (61–100)	96.6 (88–100)	0.46
Length of stay (days)		5.1 (1–70)	5.2 (1–70)	3.5 (1–38)	0.001
Non-invasive ventilation or invasive ventilation		51 (12.6%)	50 (13.6%)	1 (2.7%)^a^	0.10
In-hospital mortality		22 (5.4%)	20 (5.4%)	2 (5.4%)	1.0
Died within 5 days		14 (3.5%)	12 (3.3%)	2 (5.4%)	0.38

Patients who had arterial blood gases measured are compared to patients without arterial blood gases. Continuous variables are presented as mean and (minimum–maximum).

a Intubated before artery puncture with oxygen saturation 50% measured on pulse oximetry.

In the ambulance at arrival to the ED, a SpO_2_ above 92% was recorded in 373 of 404 (92.3%) patients. Only 15 of these patients were not treated with oxygen and information was missing in seven patients. Therefore, 352 of 397 (88.7%) patients received inappropriate oxygen therapy.

Blood gas analysis was not performed in 37 of all 405 patients (9.1%) (refused by the patient, reluctance of the staff to perform the test, or considered not indicated by the doctor). Pre-hospital data in patients with and without blood gas analysis were not statistically different (Table 1).

Hypercapnia (P_a_CO_2_>6.0 kPa) in the initial ABG analysis was seen in 234 (63.6%) of 368 patients, and 125 (34.0%) of 368 patients had respiratory acidosis (Table 2). The combination of inappropriate oxygen therapy and respiratory acidosis was seen in 33.3% of the patients (Table 3).

**Table 2:** Relationship between arterial blood gases and pH, inappropriate high concentration of oxygen delivered (SpO_2_>92% and supplemental oxygen) by the ambulance, treatment with non-invasive ventilation (NIV) or invasive ventilation (IV), and death in 368 patients, who were brought by ambulance to the emergency department

	PH	*N* (%)	*N* (%) with ‘inappropriate high concentration of oxygen therapy’	*N* (%) NIV alone	*N* (%) NIV or IV	*N* (%) in-hospital deaths
Normocapnia	Any	134 (36.4)	118/132 (88.1%)	2 (1.5%)	4 (3.0%)	2 (1.5%)
Hypercapnia (P_a_CO_2_ >6.0 kPa)	≥7.35	104 (28.3)	93/103 (90.1%)	0	0	8 (7.7%)
	7.30–7.34	52 (14.1)	48/51 (94.1%)	6 (11.5%)	7 (13.5%)	4 (7.7%)
	7.25–7.29	41 (11.1)	37/41 (90.2%)	14 (34.1%)	15 (36.6%)	3 (7.3%)
	<7.25	37 (10.1)	28/36 (77.8%)	23 (62.2%)	24 (64.9%)	3 (8.1%)

**Table 3:** Outcomes according to whether oxygen was given in the ambulance and whether the flow was appropriate or not (SpO_2_<92%) in 398 patients^a^

	No oxygen and SpO_2_>92%	Oxygen therapy and SpO_2_>92%^b^	Oxygen therapy and SpO_2_≤92%^c^
*N*	15	352	30
Respiratory acidosis, N (%)	1/12 (8.3%)	108/324 (33.3%)	13/26 (50.0%)
LOS, days (min.–max.)	1.9 (1–9)	5.1 (1–70)	6.7 (1–24)
NIV alone, N (%)	0	40 (11.4%)	5 (16.1%)
NIV or IV, N (%)	0	44 (12.5%)	6 (19.4%)
In-hospital deaths, N (%)	0	17 (4.8%)	5 (16.1%)

a Missing data on pre-hospital oxygen flow in seven patients and on SpO_2_ in one patient

b Inappropriate oxygen therapy

c appropriate oxygen therapy. NIV: non-invasive ventilation; IV: invasive ventilation.

Among 398 patients with known oxygen flow, 344 (86.4%) patients were treated with HCO (defined as ≥5 L/min on mask or nasal ≥4 L/min), and only 38 (9.5%) were treated with low-moderate concentrated oxygen (Table 4). Compared to patients treated with HCO, these patients had a tendency towards higher in-hospital mortality (13.2% vs. 5.2%; *p*=0.05). Administered oxygen concentration was not associated with length of stay (*p*=0.19) or need of ventilator support (*p*=0.80).

**Table 4:** Outcomes according to oxygen flow given in the ambulance in 397 patients^a^

	Low–moderate oxygen flow: Oxygen flow on mask: 1–4 L/min or oxygen flow nasal: 1–3 L/min			High concentration oxygen: Oxygen flow on mask: ≥5 L/min or oxygen flow nasal: ≥4 L/min^b^		
SpO_2_	<88%	88–92%	>92%	<88%	88–92%	>92%
*N*	0	5	33	10	15	318
Respiratory acidosis, N (%)		2/3 (66.7%)	7/29 (24.1%)	6/10 (60%)	5/13 (38.5%)	101/295 (34.2%)
LOS, days (min.–max.)		4.4 (1–10)	4.5 (1–30)	9.3 (1–24)	6.1 (1–21)	5.2 (1–70)
NIV alone, N (%)		1 (20%)	2 (6.1%)	2 (20%)	2 (13.3%)	38 (11.9%)
NIV or IV, N (%)		1 (20%)	3 (9.1%)	2 (20%)	3 (20%)	41 (12.9%)
In-hospital deaths, N (%)		2/5 (40%)	1 (3.0%)	0	0	11 (3.5%)

a Fifteen patients received no oxygen therapy and flow was not reported in seven patients.

b SpO_2_ is missing in one patient.

Among patients with HCO, the level of SpO_2_ (>92 or ≤92%) was not associated with length of stay (*p*=0.10), need of ventilator support (*p*=0.32), or in-hospital mortality (*p*=0.36).

**Fig. 1:**
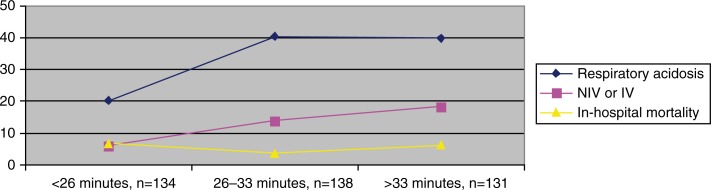
Relationship between transit time (tertiles) (*n*=403) and various outcomes: respiratory acidosis, non-invasive ventilation (NIV) or invasive ventilation (IV), and in-hospital mortality.

Only 46 (36.8%) of all 125 patients with respiratory acidosis and 39 (36.1%) of those 108 patients with inappropriate HCO and respiratory acidosis were treated with NIV or invasive ventilation. Fourteen (3.5%) of 405 patients died within 5 days, and 22 (5.5%) died during hospitalisation. Patients with inappropriate oxygen therapy had lower in-hospital mortality (4.8%) compared to patients with saturation less than 93% on oxygen (16.1%; *p*=0.011) (Table 3). Long transit time was associated with respiratory acidosis and need of ventilatory support but not with mortality (Fig. 1). Length of stay was significantly longer for patients with transit time longer than 26 min than for patients with shorter transit time (5.2 days vs. 4.8 days; *p*=0.009); however, transit time longer than 33 min was not associated with more days at hospital.

## Discussion

This large audit found that nearly all patients brought to the ED by ambulance with an exacerbation in COPD received oxygen at high flow rates, and most of the patients had inappropriate oxygen flow resulting in a SpO_2_>92%. Many patients on high flow oxygen therapy in the ambulance had respiratory acidosis on arrival to the ED – especially when transit time was long. Surprisingly, we found that patients on oxygen with SpO_2_ above 92% had a good prognosis, low need of ventilatory support, few hospital days and low in-hospital deaths.

Most patients with respiratory acidosis could be discharged a few days later without assisted ventilation. Although, we do not have data on changes in oxygen flow after the initial ABG analysis, we believe that in most cases, the respiratory acidosis was reversible after the reduction of oxygen flow. In general, the transit time to hospital in our study was short. As expected, we found a strong relationship between long transit time and presence of respiratory acidosis and the need of supported ventilation. Low SpO_2_ at arrival, but not inappropriate oxygen therapy or transit time, was associated with increased in-hospital mortality.

In our study, the proportion of patients who had SpO_2_ >92% at arrival to the ED was higher than in the audit from New Zealand (92.2% vs. 75%) (6). Frequency of inappropriate oxygen therapy has not been reported previously.

The transit time, in our study, was comparable with findings in two British studies (mean approximately 30 min) (14, 15), but shorter than in the studies from Australia and New Zealand (mean 47 and 50 min) (6, 7). Durrington et al. found that longer transit time was associated with increased complication rate (15). This is in keeping with our results, where transit time exceeding 26 min was associated with 2.0-fold increased risk of respiratory acidosis and 2.6-fold increased risk of need for ventilatory support but no increase in mortality.

The frequencies of hypercapnia and respiratory acidosis were high in our study and comparable with findings in a small Australian study of COPD patients admitted by ambulance (16). Forty-one (95%) patients were hypercapnic, and 21 (49%) were acidotic (16). In contrast, two large British studies found much lower frequencies (5, 17). In a prospective study of nearly 1,000 COPD patients admitted to hospital, 50% had hypercapnia and 20% had acidosis on admission (5). Similarly, an audit of nearly 10,000 COPD admissions found 20% with respiratory acidosis (17). In both studies, the proportion of patients brought to hospital by ambulance was not reported (5, 17), and data on pre-hospital oxygen therapy were not reported in the study by Plant et al. (5). In the large audit, ‘only’ 30% of the patients with recorded data received >35% oxygen in the ambulance prior to admission, and that could explain at least some of the differences in ABG between our study and the British study (17).

We found that our patients treated with inappropriate oxygen flow (SpO_2_>92%) had a good prognosis. Only 12.7% of the patients needed ventilatory support, 4.9% died at the hospital, and average length of stay was 5.1 days. Our study shows that patients who develop respiratory acidosis during inappropriate oxygen therapy, as a rule normalise their pH, when they receive standard treatment in the ED with target saturation 88–92%. The frequency of ventilatory support in our study is in accordance with the large British studies, where about 11% of the patients received NIV, but in-hospital mortality in our study was lower (5.5% vs. approx. 7.7%) (17).

An Australian study compared HCO flow (8–10 L/min) with titrated oxygen to achieve SpO_2_ between 88 and 92% in a randomised design. They found that patients in the HCO arm had higher mortality than those in the titrated oxygen arm (9% vs. 4%) (7). However, the authors have not provided information that could explain this difference in mortality. Treatment at the hospital is not described. Although it is a prospective study, the number of missing data is remarkably high. Only 56% of the patients in the intervention arm continued titrated oxygen, and the remaining patients were given high concentration oxygen – thus implying a bias of severity. ABG was only measured in less than 20% of those patients with confirmed COPD, and information on supported ventilation was missing in more than 20% of patients with confirmed COPD. The study hypothesis was that HCO flow results in a higher frequency of respiratory acidosis, which necessitates supported ventilation, but the frequency of supported ventilation was not statistically different in the two arms (14% vs. 10%; *p* value=0.34). The strength of our study is a nearly complete dataset including ABG on a large number of consecutive COPD patients.

Despite frequent use of high flow oxygen, inpatient mortality in our study was either comparable with the studies of Wijesinghe et al. (6), Austin et al. (7) (patients with titrated oxygen), and Joosten et al. (16) or lower than other studies by Austin et al. (7) (patients with high flow), Durrington et al. (15), and Roberts et al. (17). The average length of hospital stay for our patients was comparable with findings from Austin et al., where COPD patients with titrated oxygen were hospitalised, on average, for 5.4 days (7).

We demonstrated that administration of inappropriate oxygen flow in the ambulance does not result in an extended hospital admission, or higher need of ventilatory support, or higher mortality rate. We believe that the reason for the good prognosis of our patients is early detection of patients sensitive to oxygen with ABG measurement and titrated oxygen therapy in the ED. We cannot rule out that the prognosis could be even better, if a lower pre-hospital oxygen flow was used. Whilst it seems reasonable to use titrated oxygen flow when COPD patients are brought to hospital by ambulance, we need more evidence.

### Limitations

This is a retrospective study. Only the first blood gas analysis was registered, so it is not possible to provide information on changes in blood gases over time.

## Conclusions

The vast majority of patients acutely admitted for acute exacerbation of COPD received inappropriate oxygen therapy in the ambulance, but their need for ventilatory support and mortality were low. More randomised studies are needed to clarify the optimal pre-hospital oxygen therapy in patients with exacerbations of COPD.
